# Dual Targeting of Soluble Oligomeric and Aggregated Transthyretin with a Monoclonal Antibody Ameliorates Experimental Neuropathy

**DOI:** 10.3390/biology11101509

**Published:** 2022-10-15

**Authors:** Michael Fassler, Sagi Tshori, Yaron Barac, Dawn E. Bowles, Clara Benaim, Jacob George

**Affiliations:** 1 Heart Center, Kaplan Medical Center, Rehovot, Hebrew University of Jerusalem, Jerusalem 91905, Israel; 2 Cognyxx Ltd., Tel Aviv, Israel; 3 Research Authority, Kaplan Medical Center, Rehovot, Hebrew University of Jerusalem, Jerusalem 91905, Israel; 4 The Division of Cardiovascular and Thoracic Surgery, Rabin Medical Center, Petach Tikva 4941492, Israel; 5 Surgical Sciences Division, Department of Surgery, Duke University, Durham, NC 27710, USA

**Keywords:** ATTR amyloidosis, monoclonal antibody, immunotherapy, neuropathy

## Abstract

**Simple Summary:**

Amyloid Transthyretin (ATTR) amyloidosis results from the aggregation of liver-derived transthyretin within the heart and nervous system. Herein, we further define the properties of our recently described monoclonal antibody targeting misfolded TTR. We show that the antibody binds both soluble as well as aggregated insoluble forms of TTR, thus addressing two triggering forms of the protein independently mediating neurotoxicity. Furthermore, we show that the antibody enhances the clearance of aggregated TTR via microglia and protects neurons from misfolded-TTR-mediated toxicity. These two complementary protective mechanisms are evident by the ability of the antibody to protect from experimental neuropathy inducible by misfolded TTR infiltrated into the sciatic nerves of rats. The findings of the study suggest that the approach of amyloid debulking may stand as a promising strategy for disease modification in patients with ATTR polyneuropathy.

**Abstract:**

ATTR amyloidosis comprises a spectrum of multiple clinical presentations, including, predominantly, neuropathy and cardiomyopathy. The common triggering pathogenic protein is misfolded transthyretin, a carrier protein that destabilizes misfolds and assembles into mature amyloid fibrils. The current management of ATTR amyloidosis includes the use of agents that stabilize TTR or attenuate its liver inducible production. Herein, we tested the hypothesis that a monoclonal antibody targeting the soluble oligomeric as well as the aggregated TTR would influence experimental neuropathy. We have shown that Ab-A, our previously described humanized IgG monoclonal antibody, dose-dependently ameliorates the toxicity to neurons triggered by misfolded TTR oligomers. Furthermore, the antibody that exhibits wide misTTR epitope recognition that includes the oligomeric and aggregated forms of the protein dose-dependently enhances the uptake of misfolded TTR to microglia, the resident predominant cells of the innate immune system within the CNS. These in vitro mechanistic properties of the antibody were corroborated by experimental in vivo data showing that the antibody rapidly clears human TTR amyloid extracts infiltrated to the sciatic nerves of rats. Thus, the monoclonal antibody targeting soluble and aggregated TTR is effective in experimental neuropathy, likely due its ability to act as a neuroprotective agent, as well its misTTR-mediated clearance via microglia.

## 1. Introduction

Transthyretin (TTR) is a 55 kD protein predominantly synthesized in the liver that acts as a carrier of the thyroid hormone and the Retinol binding protein [1,2]. The requisite role of TTR in the maintenance of hemostasis is uncertain. However, when misfolded, TTR can produce a spectrum of pathologies that involve multiple organ systems [3].

Over 140 mutations have been found with regard to TTR, mostly increasing its propensity to destabilize, misfold and aggregate into mature amyloid fibrils that predominantly deposit in the central nervous system and the heart, producing the respective clinical syndromes of polyneuropathy and cardiomyopathy [3].

Whereas mutated TTR causes hereditary transthyretin amyloidosis ATTRv, a rare disease with a devastating outcome, native non-mutated TTR can nevertheless undergo spontaneous aggregation that results in a predominant cardiac phenotype of wild type ATTR cardiac amyloidosis with consequent heart failure that tends to co-occur with neural involvement in many cases [4].

The processes that follow TTR misfolding, culminating in the organization into mature, Congo red positive amyloid fibrils, are not yet adequately understood, and this ambiguity relates to the role of intermediate species comprising soluble oligomers versus fibrils in the pathogenesis of the phenotype of ATTR amyloidosis [5].

In recent years, the breakthrough in understanding the pathogenesis of ATTR amyloidosis led to a proliferating pipeline of drugs based on the stabilization of the TTR protein as well as silencing its production using anti-sense technologies, RNA inhibitors and gene-editing drugs [4,6]. Although all these approaches can be regarded as disease-modifying, they are given at stages where patients are symptomatic and considerable amyloid burden is already present in the CNS and the heart. By definition, all of these agents act by delaying the accumulation of further misfolded TTR and, thus, are likely to be mostly effective in the early stages of pathology, as they do not act on the mature amyloid fibril.

The concept of amyloid plaque removal, which has been tested in experimental animal models, addresses the preexisting amyloid fibrils and thus has the theoretical advantage of acting in patients with more advanced disease [7,8,9]. The use of monoclonal antibodies targeting misfolded TTR, thereby leading to Fc gamma-mediated clearance, is the most promising approach that would have to gain clinical proof of concept in the future. We have previously generated an antibody that targets misfolded forms of TTR that was capable of ameliorating experimental TTR-mediated myocardial dysfunction [8].

In this paper, we describe the unique dual mechanism of action of our recently developed anti-TTR IgG monoclonal antibody [8] and its application as a neuron-protecting agent from misfolded TTR-mediated toxicity, as well as its plaque debulking properties in a model of sciatic nerve amyloidosis.

## 2. Materials and Methods

### 2.1. Uptake of Aggregated TTR in Microglia Cells

BV-2 (mouse microglia, ICLC ATL03001) cells were split into 12-well culture plates (Greiner CELLSTAR multiwell culture plates) on the day before the experiment. A total of 0.3 μM aggregated TTR conjugated with Alexa 488, pre-incubated with 0.1 0.2, 0.5, 1, 2, 3 and 4 μg/mL of Ab-A or 0.1, 1, 3 and 4 μg/mL human control IgG for 2 h at room temperature, were added to cells with culture media (RPMI 1640, 10% FBS, 2 mM L-Glutamine, 1% Pen/Strep). The conditioned medium was also pre-incubated with Ab-A (without aggregated TTR) for 2 h as a control. The cells were then incubated at 37 °C for 24 h and harvested. Extracellular and cell surface-aggregated TTR conjugated with Alexa 488 in cell pellets was quenched by the incubation of 0.4% trypan blue in PBS (pH 4.4) for 1 min. Flow cytometry (FL1—blue laser (488 nm)) was used to measure aggregated TTR (conjugated to ALEXA fluor 488) uptake, indicated as the relative geomean fluorescence intensity (MFI).

### 2.2. Neuronal Toxicity Assessment Employing Aggregated TTR and the Effect of Ab-A

The toxicity of oligomeric TTR was assessed by cell titer 96 proliferation assay (Promega, G3580) on differentiated neuronal SH-SY5Y cells (ATCC, CRL-2266). For the cell titer 96 proliferation assay, the cells were plated at a density of 5000 cells per well on 96-well plates in 100 μL of culture medium per well. Following 72 h, the medium was exchanged with 100 μL of fresh medium containing 1% FBS and 10 μM of retinoic acid (RA) for neuronal cell differentiation. After seven additional days, 5 μM of oligomeric TTR was incubated with Ab-A for an additional 1 h at 37 °C with shaking and added to the differentiated neuronal cells for 24 h at 37 °C. Control samples were prepared with the addition of identical volumes of buffer. After 24 h of incubation, 20 μL of CellTiter 96 Aqueous One Solution (Promega) were added in each well, and the samples were incubated at 37 °C. The absorbance was measured at 570 nm in a Tecan infinite 200 pro microplate reader (Tecan, Männedorf, Switzerland) after 4 h.

### 2.3. Assessment of Clearance of Aggregated TTR in a Model of ATTR Polyneuropathy Ab-A

WT Rats (*n* = 9) were anesthetized by Isoflurane before their legs were opened for the intraneural injection of fluorescent labeled (Alexa Fluor^®^ 647) aggregated TTR V30M [10] complexed with either Ab-A or control IgG to the sciatic nerve. Matrigel matrix (Corning) was added to 3.3 μg of aggregated TTR and either 10 μg Ab-A antibody or control IgG in PBS in a 1:1 volume ratio to a final 50 μL volume. The control animals received sterile PBS and Matrigel (1:1) with no TTR. A single needle insertion into the left or right sciatic nerve of the rat was used to spread the aggregated TTR and treatment complex. Immediately after surgery (t = 0) and 24 h later (t = 24 h), the animals underwent fluorescence imaging for TTR detection. Sciatic nerve retention was assessed quantitatively to calculate right and left sciatic nerve fluorescence by drawing regions of interest in each side.

### 2.4. Von Frey Test

The rats were placed individually in a small cage on a wire mesh surface. This standard mechanical sensitivity test comprises calibrated plastic filaments applied to the plantar surface of the hind paw of the rat.

### 2.5. Animals

WT rats (Sprague–Dawley) were provided by Envigo RMS (Israel), Ltd. (Jerusalem, Israel). All housing, breeding and procedures have been reviewed and approved by “The Israel Board for Animal Experiments” and were in compliance with “The Israel Animal Welfare Act” under the #IL20-8-368 protocol. The animals were maintained in the local institutional animal house with a 12 h light and 12 h dark cycle.

### 2.6. Preparation of Aggregated and Oligomeric TTR

Lyophilized purified recombinant TTR monomer or recombinant TTR V30 M (1 mg Alexotech, Sweden) was thawed at room temperature for 10 min, followed by the addition of 0.5 mL of sterile PBS to a final concentration of 2 mg/mL. TTR was transferred to a new low-binding, sterile 1.5 mL microcentrifuge tube (Protein LoBind Tube 1.5, Eppendorf tubes, Cat no.: 022431081), followed by the addition of 500 μL TTR aggregation buffer ×2 (20 mM sodium acetate (pH 4.3), 200 mMKCl and 20 mM EDTA) to generate aggregated or oligomeric TTR in a final volume of 1 mL and at a final concentration of 1 mg/mL. The tube was placed in 37 °C incubation for 72 h (maximum 4 days) for aggregated TTR [11]. This procedure generated thioflavin T-positive fibrils, detectable in dynamic light-scattering particle size analysis (Worcestershire, UK) [8]. Water-soluble TTR oligomers were prepared with the above protocol and by limiting the aggregation time of TTR to 4 h at 37 °C. Oligomers (soluble fraction of the solution) were characterized by SDS-PAGE and size-exclusion chromatography on Superdex 200 columns (Hiload Superdex 200, GE Healthcare, Chicago, IL, USA). The solution was divided into aliquots in sterile microcentrifuge tubes and stored at −80 °C before use.

### 2.7. Conjugation of Aggregated TTR with Alexa Fluor 488/647

Aggregated TTR and aggregated TTR V30 M were labeled with Alexa Fluor 488 (Alexa Fluor^®^ 488 Labeling Kit Lightning-Link, Cat no.: ab236553, Thermo Fisher Scientific) and Alexa Fluor 647 (Alexa Fluor^®^ 647 Labeling Kit Lightning-Link, Cat no.: ab269823), respectively, as described in the manufacturer’s protocol. All materials and reagents were equilibrated to room temperature prior to use. Labeling kit modifier reagent was added (10% *v/v*) to the aggregated TTR to be labeled, mixed gently and incubated for 15 min. Prepared protein mixture was added to the Alexa Fluor 488/647 conjugation lyophilized mix, resuspended gently and incubated for 15 min or longer in the dark (20–25 °C). Quencher reagent (10% *v/v*) was added to the conjugated protein to deactivate the excess unbound label post-reaction.

### 2.8. Dot Blot Analysis

Tissue samples used for this study were procured by the Duke Human Heart Repository (DHHR). The DHHR is a Duke University Hospital System Institutional Review Board-approved tissue repository. Samples were obtained using written informed consent or a waiver of consent for discarded tissues. All samples used in this study were de-identified, with all Health Insurance Portability and Accountability Act (HIPAA) identifiers removed. Left ventricular (LV) cardiac tissue from the heart failure subjects was obtained at the time of left ventricular assist device (LVAD) implantation or heart transplantation. At the time of collection, the LV tissue was sharply dissected and then flash frozen in a cryovial using liquid nitrogen. The tissue was then stored at −80 °C until it was utilized. Subject data from the time of surgery were retrospectively collected by DHHR personnel from an electronic medical record database. These samples were provided under IRB Protocol # Pro00005621. Two μg-soluble TTRs from human heart extracts from each sample (DHHR identifiers #836, #802, #1039) were loaded into 0.2 μm nitrocellulose membranes until they were dry. The membranes were blocked with 5% dry milk in TBST incubated at room temperature for 1 h and then incubated at 4 °C for 12 h with 1 μg/mL of Ab-A or human control IgG as the primary antibodies, diluted in 1% BSA/TBST. The membranes were washed three times with TBST, followed by goat anti human conjugated to HRP (1:10,000) incubation for an additional 1 h incubation at room temperature. The membranes were developed using a Fusion Solo 7S imager system (Vilber, France).

### 2.9. Aggregated TTR Extraction from Tissues

ATTR fibril aggregates were extracted from the frozen cardiac tissues obtained at autopsy and semi-purified using the detergent-free protocol described by Pras et al. [12]. The amyloid conformation was preserved by relying on the partial solubility of amyloid fibrils in pure water.

### 2.10. Measurement of Ab-A Binding Affinity to Native TTR, Oligomeric TTR and Aggregated TTR

ELISA plates were coated with 100 µL/well of 1.5 µg/mL aggregated TTR and incubated overnight at 4 °C. The wells were aspirated and washed three times (300 µL/well) with washing buffer (0.1% TBST), followed by a 2 h incubation at room temperature with 300 µL/well blocking buffer (5% milk/TBST) with gentle shaking. The plates were then washed three times and incubated for 1 h at room temperature with 100 µL/well of Ab-A (0.6 µg/mL) with different concentrations of native TTR, oligomeric TTR or aggregated TTR complexes (competition) diluted in sample buffer (0.5% milk/TBST). After additional washing steps, the HRP-labeled goat anti human antibody (1:10,000) was added to the wells (100 µL/well) and incubated for 1 h at room temperature. Development was performed with a substrate reagent containing tetramethylbenzidine and hydrogen peroxide (R&D systems, Minneapolis, MN, USA cat # DY999).

### 2.11. Statistical Analysis

The values shown in the figures are presented as either the mean +/− SD or the mean +/− SEM, as indicated. *p* values were calculated by the paired two-tailed Student’s t test. Statistical analysis was performed using Prism 8. *p* < 0.05 was defined as the significance.

## 3. Results

**Figure 1:**
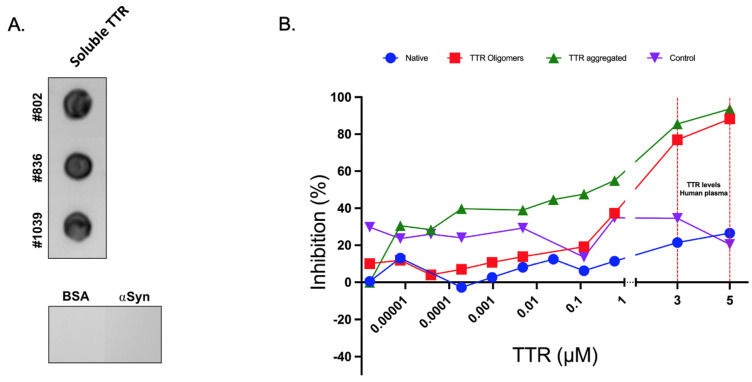
Binding of Ab-A to native, oligomeric and aggregated TTR. (**A**) Ab-A was tested for binding purified soluble TTR from human heart extract. Dot blot of mAb-A antibody to soluble TTR. BSA and alpha synuclein were blotted as negative control. (**B**) Binding of Ab-A to TTR Native, Soluble TTR oligomers or aggregated TTR and the consequent interaction inhibition of Ab-A to the aggregated TTR. BSA red lines illustrate the levels of endogenous TTR in human plasma. One representative result out of three is shown.

We used a dot blot immunoassay to verify the binding of the Ab-A antibody to the purified soluble TTR from the human heart extracts (Figure 1A). Ab-A bound the soluble TTR oligomers from the human heart extracts of the three human ATTR patient samples. In contrast, no detectable binding of Ab-A to the negative control proteins BSA and *α*-Synuclein was evident.

In order to analyze and compare the affinity of Ab-A to native TTR, soluble TTR oligomers or aggregated TTR, a standard competition ELISA assay, was performed using aggregated TTR for solid phase coating. A higher inhibition was observed when using aggregated TTR and TTR oligomers as competitors than that using native TTR or BSA (Figure 1B). Therefore, we conclude that Ab-A binds with a higher affinity to aggregated TTR and to soluble TTR oligomers compared to native TTR or BSA and that the binding is dose-dependent.

**Figure 2:**
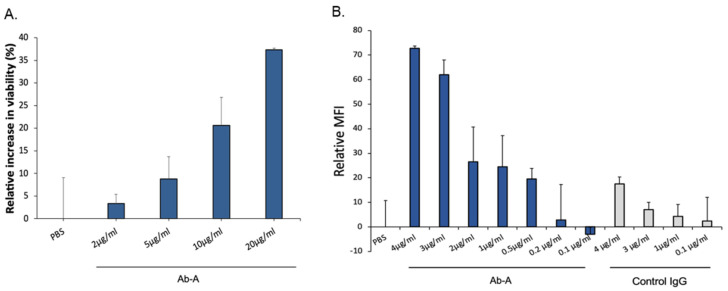
The effect of Ab-A on SH-SY5Y neuronal cell viability after oligomeric TTR-induced toxicity and on the uptake of aggregated TTR by microglia. (**A**) The effect of Ab-A on the viability of neuronal cells (SH-SY5Y cell line) exposed to 5 µM oligomeric TTR and either PBS or increasing doses of Ab-A. The y-axis represents the relative increase in cell viability resulting from treatment with Ab-A antibody as compared to treatment with PBS. Samples were measured in quadruplicate (mean ± standard deviation). (**B**) Ab-A increases the uptake of fluorescent-aggregated TTR by mouse microglia BV2 cells. The y-axis represents the relative median fluorescence intensity (MFI) measured by flow cytometry. Samples were measured in triplicate (mean ± standard deviation). One representative result out of three is shown.

Since oligomeric TTR has a toxic effect on differentiated neuronal cells, we used a cell viability assay to study the protective role of Ab-A (Figure 2A). Neuronal cells were incubated with oligomeric TTR in the presence of either increasing concentrations of Ab-A or PBS. A dose-dependent increase in cell viability was observed. Neuronal cells were incubated with oligomeric TTR and with either PBS or 2 μg/mL, 5 μg/mL, 10 μg/mL or 20 μg/mL Ab-A. Compared to PBS, cell viability was increased by 3.3% ± 2.1, 8.8% ± 4.9, 20.6% ± 6.2 and 37.3% ± 0.3, respectively. Therefore, a dose-dependent Ab-A protection of neuronal cells from TTR toxicity was observed.

Aggregated TTR was conjugated to Alexa fluor-488, and the uptake of aggregated TTR by mouse microglia cells was measured by flow cytometry (Figure 2B). The addition of Ab-A increased the cellular uptake of aggregated TTR, with a maximum uptake at 4 μg/mL (maximum relative MFI = 72.8 ± 1.0), while a low uptake was observed after the addition of control IgG (maximum relative MFI = 17.5 ± 2.8). Increasing concentrations of Ab-A resulted in an enhanced uptake of aggregated TTR by the microglia cells.

**Figure 3:**
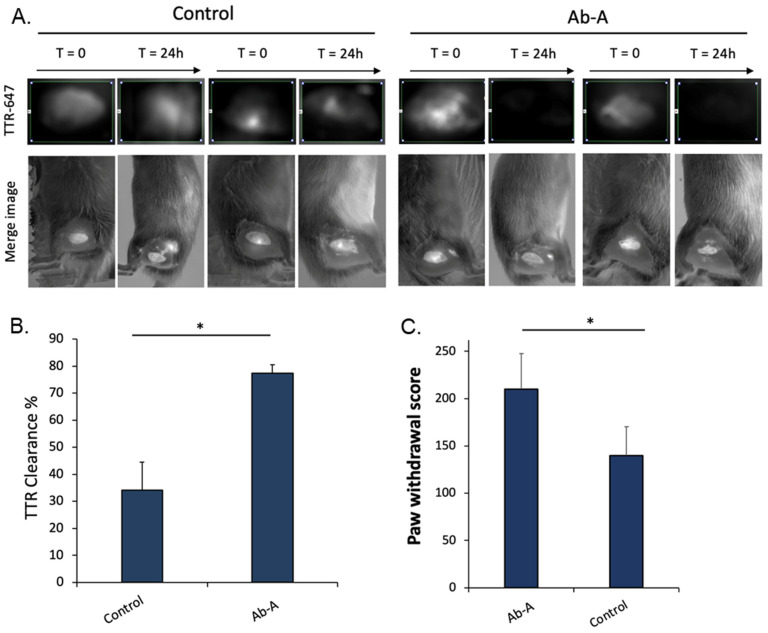
Ab-A produces the robust clearance of aggregated TTR and reduces neurologic damage in a model of ATTR polyneuropathy. (**A**) Aggregated TTR detected in rats by fluorescence imaging representative images. The rats in the left panel were treated with control IgG, and the rats in the right panel were treated with Ab-A. In each treatment group, there are two representative images showing TTR fluorescence before (T = 0) and after (T = 24 h) treatment. (**B**) Data from the image analysis of fluorescence imaging images quantitating TTR fluorescence in rats that received fluorescent TTR sciatic nerve injections and were treated with Ab-A or control IgG treatments. The *y*-axis represents the % clearance of TTR measured by fluorescence detection in the rat’s tissue 24 h after surgery (* *p* = 0.008). (**C**) Von Frey paw withdrawal score test performed 10 days post-operation in rats injected with aggregated TTR into the sciatic nerve, with and without Ab-A treatment (* *p* = 0.022). Results are presented as the mean ± SEM.

The TTR polyneuropathy model was used to detect the sciatic nerve deposition of the aggregated mutated recombinant V30M TTR protein with fluorescence imaging (Figure 3). Rats were injected with aggregated V30M TTR protein and with either the control IgG or Ab-A antibody. The rats underwent fluorescence imaging immediately (T = 0) and after 24 h (T = 24 h). Fluorescence was more profoundly decreased after 24 h in the Ab-A group than in the control IgG group (Figure 3A). Fluorescence levels were measured both immediately and after 24 h, and TTR clearance was calculated. TTR clearance from the Ab-A-treated sciatic nerve was 77.4% ± 3.0 compared to 34.1% ± 10.4 from the control IgG-treated sciatic nerves (*p* = 0.008). This result demonstrates that Ab-A clears and degrades aggregated TTR in the sciatic nerve of rats.

Rats from the TTR polyneuropathy model were tested 10 days post-operation for mechanical allodynia using the Von Frey paw withdrawal test (Figure 3C). The paw withdrawal score was 210 ± 37.6 in the Ab-A-treated sciatic nerve side compared to 140 ± 30.2 in the untreated (no antibody) sciatic nerve side (*p* = 0.022), indicating less damage to the sciatic nerve with the Ab-A treatment.

## 4. Discussion

In the current work, we demonstrate the unique binding features of the anti-misTTR Ab-A and corroborate these characteristics with the protective properties that are observed in a series of in vitro studies and in a model of inducible TTR neuropathy.

TTR misfolds into soluble oligomers and amyloidogenic monomers that subsequently assemble into protofibrils and mature amyloid fibrils. Whereas the intuitive view of the pathogenesis would be that mature amyloid compresses the nerves and the heart to produce the respective phenotypes, accumulating data suggest that soluble intermediates including oligomers can produce toxicity to the neurons and cardiomyocytes, thereby contributing to the clinical presentation of the disease [13,14]. These reports are in line with multiple studies showing that soluble beta amyloid oligomers are more toxic to neurons than mature amyloid plaques and could be more detrimental with regard to the clinical outcome [15,16]. This has led to considerable and advanced efforts to target these soluble oligomers rather than mature amyloid plaques in Alzheimer’s disease [17,18].

Ab-A was developed to produce a wide misfolded TTR coverage so as to include binding to soluble oligomers and mature amyloid fibers. To be able to prove this concept, we have made specific competitive inhibition tests. The direct binding of native TTR to solid phase surfaces such as ELISA plates and Surface Plasmon Resonance chips involves alterations in the structure of TTR, and, thus, the true binding properties of the antibody cannot be assayed with accuracy. For this purpose, we employed the oligomers and aggregates of TTR as inhibitors in the fluid phase binding assays. As can be observed, oligomeric and aggregated but not native TTRs were capable of inhibiting the binding of Ab-A to extracts of ATTR amyloid from patients, suggesting the dual targeting of the antibody.

As TTR oligomers have been demonstrated to produce direct neurotoxic effects that could be related to the clinic-pathological features in ATTR amyloidosis [5,8], we wished to explore the neuroprotective properties of Ab-A in this context. Indeed, Ab-A1 has shown a dose-dependent efficacy in attenuating TTR oligomeric-mediated toxicity to human neurons in vitro. These effects are complimentary and similar to the protective effects we have observed with regard to misTTR-mediated cardiomyocyte toxicity [8].

The pathogenic hallmark of ATTR amyloidosis is the amyloid fibrils that produce a “mass effect”, compressing resident neurons and cardiomyocytes and producing neuropathy and cardiomyopathy with preserved cardiac function. Ab-A was thus tested for its ability to promote the uptake of aggregated TTR to microglia. The antibody enhanced the Fc gamma-mediated engulfment of aggregated labeled TTR to microglia that represent the major innate immune component of the CNS, in a dose-dependent manner. This effect was consistent with the previously observed effects of Ab-A with regard to the uptake of aggregated TTR to macrophages [8].

We next investigated whether the dual mechanisms of the protection of mis-TTR toxicity and enhanced microglial uptake afforded by Ab-A would translate to a beneficial effect in an animal model that would reflect both activities and would simulate human ATTR neuropathy. For this purpose, we generated a rat model where fluorescent labeled human derived TTR amyloid extracts were infiltrated into the sciatic nerves of wild type rats. We have been able to show that treatment with Ab-A but not a control isotype led to a significantly more rapid amyloid removal. This effect was corroborated by functional motoric leg tests showing the beneficial effect of the antibody over the control IgG.

The collective message from the in vitro and in vivo studies is thus that Ab-A has the unique dual mode of action of neuroprotection and amyloid debulking, which translates into improvement of the motoric function in a model of neuropathy inducible by human TTR amyloid extract. These results extend our previously published results [8], showing that this antibody protects against TTR-mediated myocardial damage to include the protection afforded by the direct inhibition of toxicity to neurons and the microglia-mediated enhanced uptake/clearance of misTTR. Current disease-modifying agents in ATTR polyneuropathy include stabilizers and silencers [19]. However, the devastating nature of this rapidly progressive disease and the lack of an effect of these agents in addressing preexisting amyloid fibrils call for an effective treatment that will engage in direct neuroprotection and plaque removal as an additive mechanism. Such combination therapies may stand as the most effective means of not only halting disease progression but also reversing its development. Although initial clinical trials are ongoing, no clinical readouts have been published yet, and safety issues have also not been reported.

## 5. Conclusions

Ab-A is a unique humanized IgG monoclonal antibody that has shown specific and wide epitope coverage to include soluble and aggregated TTR. Ab-A protects neurons from oligomeric TTR-inducible toxicity and enhances the uptake of aggregated TTR by microglia, leading to the attenuation of motoric deficit in a model of neuropathy produced by the sciatic infiltration of human-derived TTR amyloid. This approach appears attractive and complementary to the current therapeutic approaches that induce TTR stabilization and silencing.

## Data Availability

The data of the study cohort may be requested from the corresponding author.
